# Supplementary material for: Drp1 SUMO/deSUMOylation by Senp5 isoforms influences ER tubulation and mitochondrial dynamics to regulate brain development
Source: iScience. 2021 Dec 10;24(12):103484. doi: 10.1016/j.isci.2021.103484 (PMC8710555; doi:10.1016/j.isci.2021.103484)
Supplement: Document S1. Figures S1–S5 [file mmc1.pdf]

iScience, Volume *24*

Supplemental information

Drp1 SUMO/deSUMOylation by Senp5 isoforms influences ER tubulation and mitochondrial dynamics to regulate brain development

Seiya Yamada, Ayaka Sato, Naotada Ishihara, Hiroki Akiyama, and Shin-ichi Sakakibara

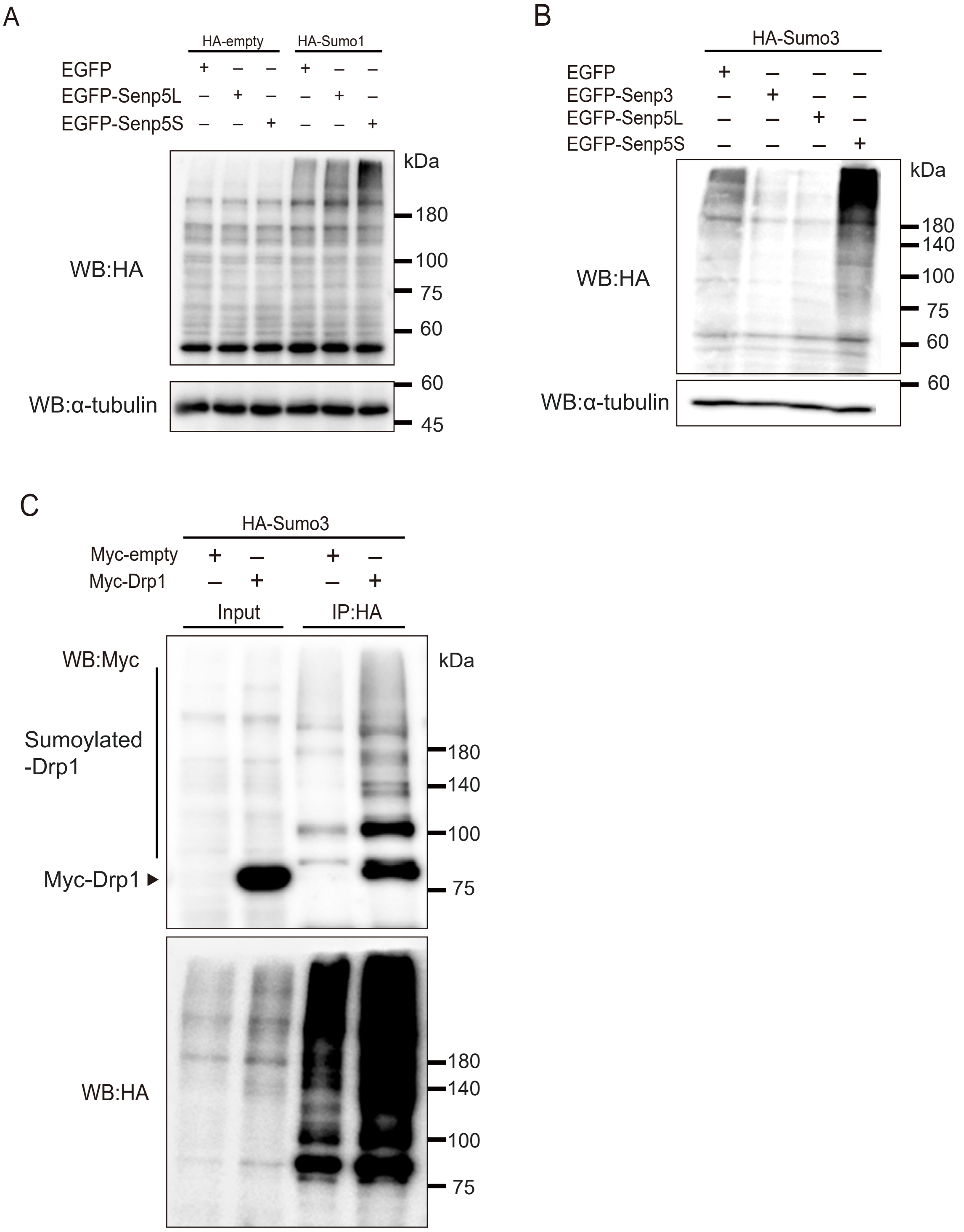

Figure S1. Senp5S promotes SUMOylation (Related to Figure 2)
(A) HEK293T cells were transfected with EGFP, EGFP-Senp5L, or EGFP-Senp5S along with HA-empty vector or HA-SUMO1, followed by immunoblotting with anti-HA (upper panel) or anti-α-tubulin (bottom panel).
(B) Neuro2a cells were transfected with EGFP, EGFP-Senp3, EGFP-Senp5L, or EGFP-Senp5S along with HA-SUMO3, followed by immunoblotting with anti-HA (upper panel) or anti-α-tubulin (bottom panel).
(C) HEK293T expressing Myc empty vector, or Myc-Drp1 along with HA-SUMO3 were immunoprecipitated (IP) with anti-HA antibody. Total cell lysates (input) or IP samples were analyzed by immunoblotting with anti-Myc (upper panel) and anti-HA (bottom panel).

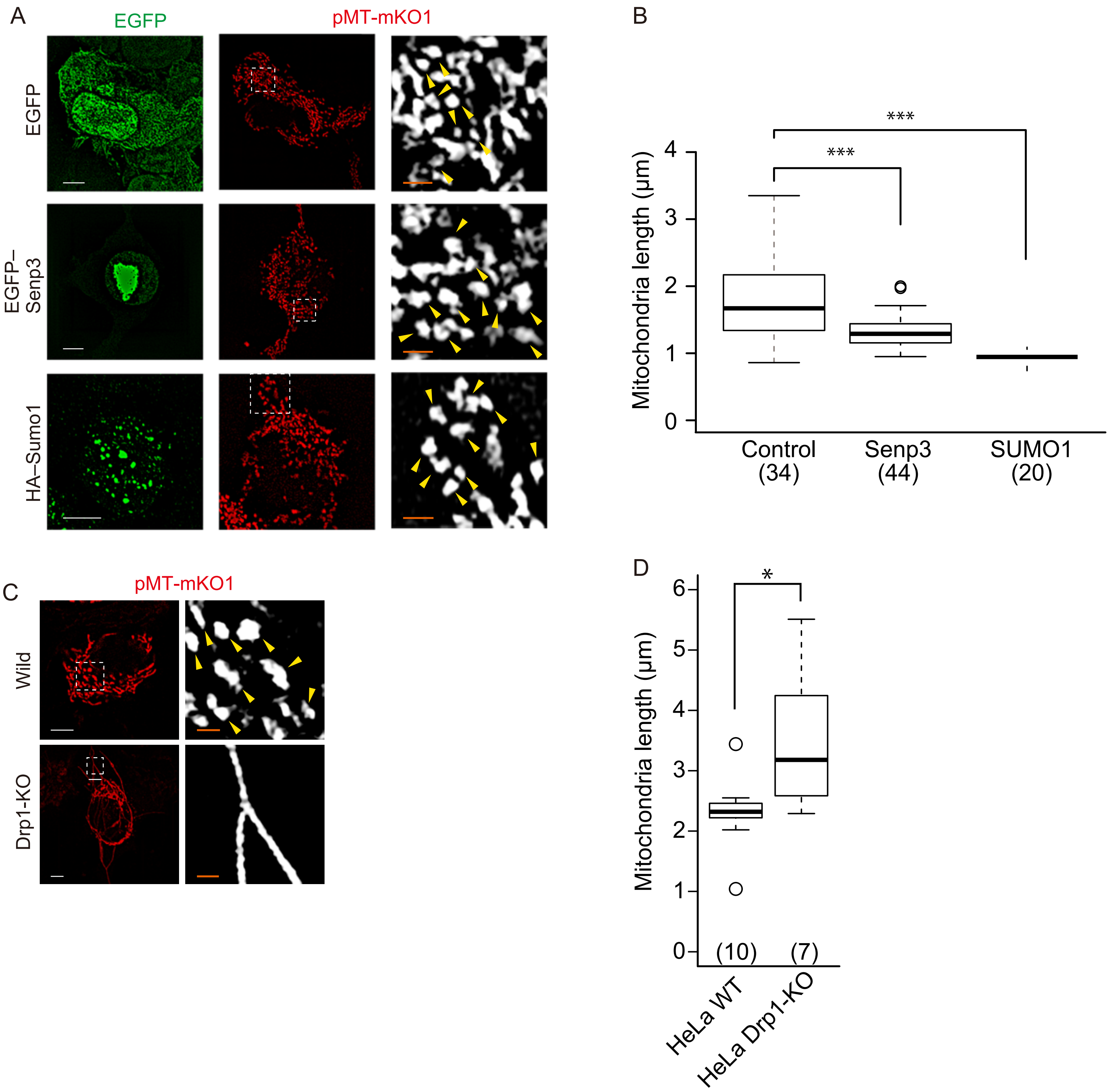

Figure S2. SUMO conjugation to Drp1 adjusts mitochondrial dynamics (Related to Figure 3)
(A, B) HEK293T cells were co-transfected with EGFP-Senp3 or HA-SUMO1 together with pMT-mKO1, followed by immunocytochemistry with an anti-HA (green). Higher magnification of the boxed areas shows pMT-mKO1+ mitochondrial morphology (white). Arrows (yellow) denote fragmented mitochondria. (B) The box and whisker plots summarize the mitochondrial length (μm). Numbers in parentheses indicate the numbers of cells measured. ***, $P < 0.001$; Welch's t-tests with Holm-Bonferroni correction.
(C, D) Confocal images of wild-type or Drp1-KO HeLa cells. pMT-mKO1 (red) was transfected to visualize mitochondrial morphology. Right column, magnified views of the boxed areas showing individual mitochondria (gray-scale). Arrowheads indicate fragmented mitochondria. (D) Box and whisker plots summarize the mitochondrial length (μm). Numbers in parentheses indicate the numbers of cells measured for mitochondrial length determination. *, $P < 0.05$; Welch's t-test. Scale bars, 5 μm in (A) and (C), and 1 μm in the magnified view.

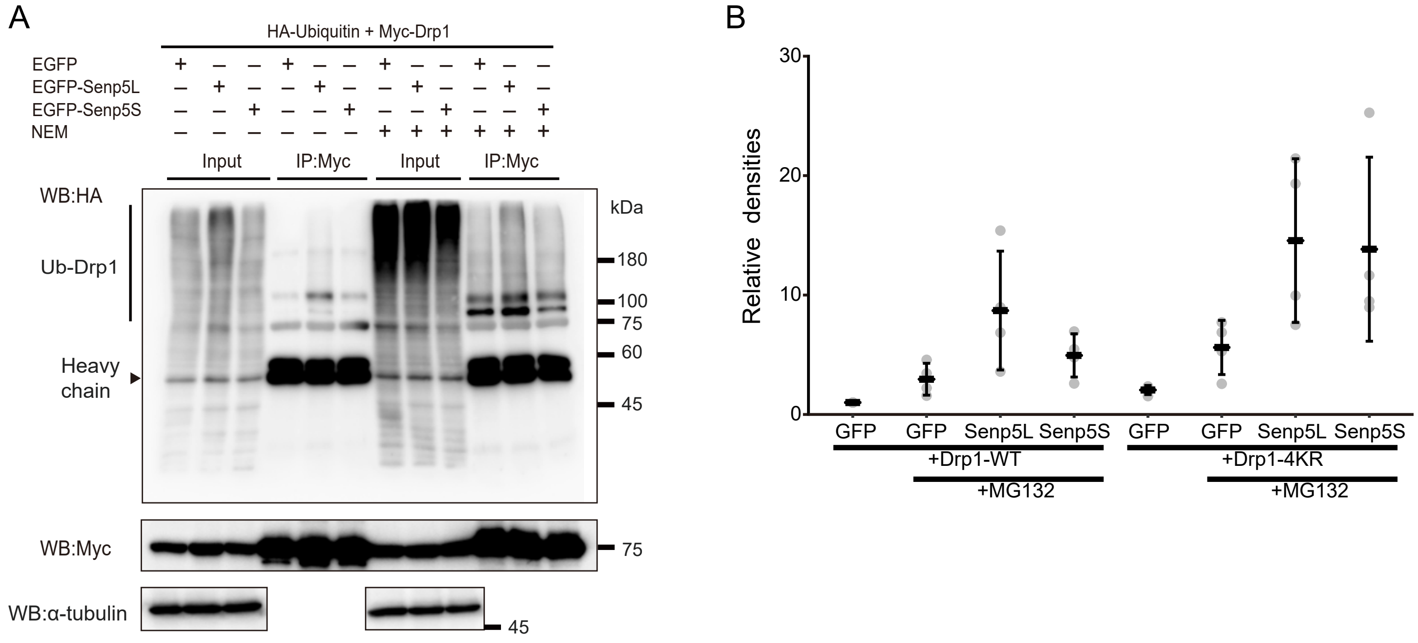

Figure S3. Drp1 deSUMOylation promotes NEM dependent ubiquitinations (Related to Figure 4) (A) HEK293 cells expressing EGFP, EGFP-Senp5L, or EGFP-Senp5S together with Myc-Drp1 and HA-SUMO3 were subjected to immunoprecipitation with an anti-HA antibody with ± N-ethyl-maleimide (NEM), followed by immunoblotting with anti-HA or anti-Myc antibodies. (B) Quantified comparison of the effect of EGFP, Senp5L, or Senp5S along with Drp1-WT or Drp1-4KR on Ub-Drp1 in figure 4A. Total chemiluminescent densities above 100kDa were measured and normalized by that of control, i.e., EGFP and Drp1-WT in the absence of MG132. Gray dots represent four independent experiments. Mean ± SD are also shown.

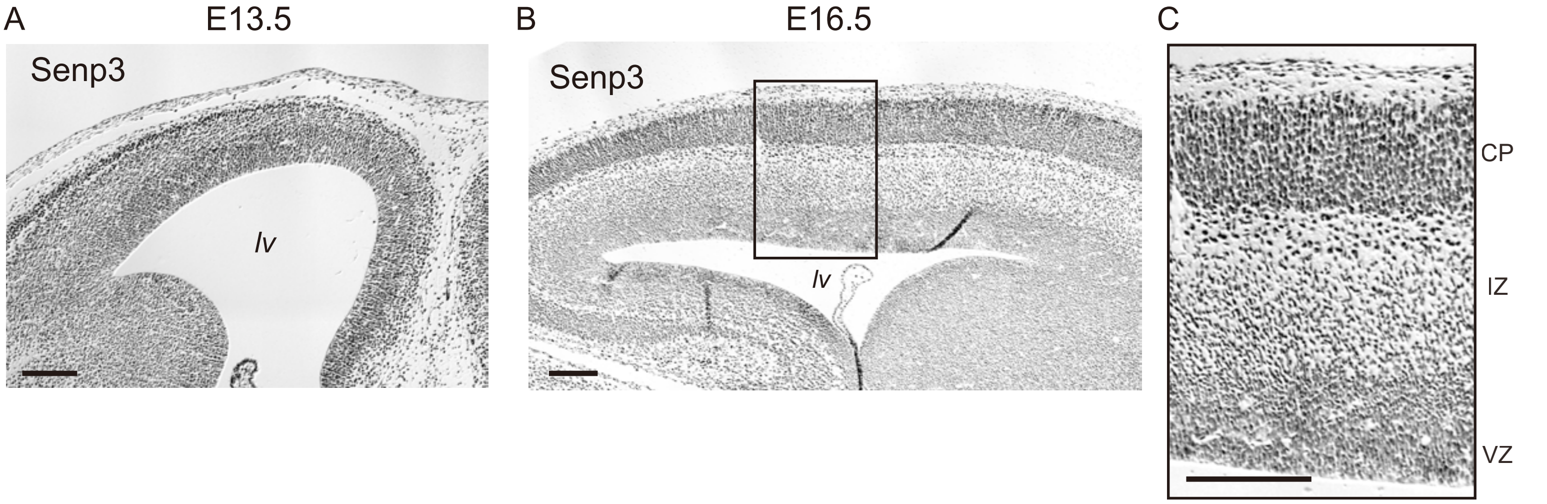

Figure S4. Senp3 expression in the developing cortex (Related to Figure 5)
(A–C) Coronal sections of the cerebral cortex at E13.5 (A) and E16.5 (B) immunostained with an anti-Senp3 antibody. (C) Higher magnification of the area of the cerebral cortex surrounding the lateral ventricle (lv) in B. Scale bars, 100 μm in (A, B); 25 μm in (C). lv, lateral ventricle; CP, cortical plate; IZ, intermediate zone; VZ, ventricular zone.

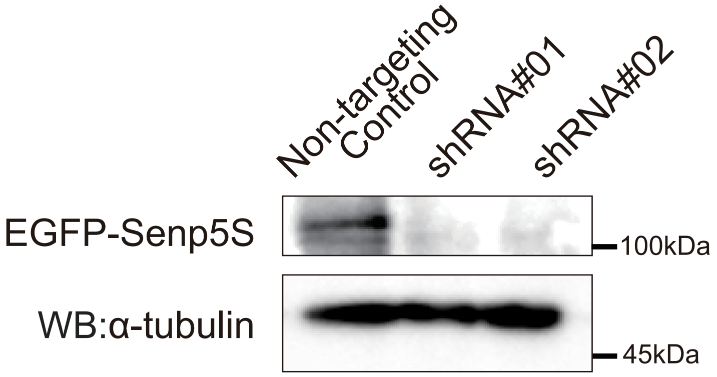

Figure S5. Validation of Senp5 shRNAs against Senp5 (Related to Figure 5)
Neuro2a cells were transfected with pCAG-EGFP-Senp5S and two different mouse Senp5 shRNA constructs (shRNA #01 and shRNA #02) or a non-targeting control shRNA, followed by immunoblotting with an anti-GFP or anti-α-tubulin antibody.
